# Facebook use and its effects on the life of health science students in a private medical college of Nepal

**DOI:** 10.1186/s13104-016-2186-0

**Published:** 2016-08-02

**Authors:** Rajesh Kumar Jha, Dev Kumar Shah, Sangharshila Basnet, Keshab Raj Paudel, Phoolgen Sah, Ajit Kumar Sah, Kishor Adhikari

**Affiliations:** 1 Department of Pharmacology, Chitwan Medical College Teaching Hospital, Bharatpur, Nepal; 2 Department of Physiology, Chitwan Medical College Teaching Hospital, Bharatpur, Nepal; 3 School of Pharmacy, Chitwan Medical College Teaching Hospital, Bharatpur, Nepal; 4 School of Public Health and Community Medicine, Chitwan Medical College, Bharatpur, Chitwan, Nepal

**Keywords:** Academic, Social networking site, Family and friend, Adverse health effects

## Abstract

**Background:**

Facebook, a popular social networking site, has been used by people of different ages and professions for various purposes. Its use in the field of medical education is increasing dramatically. At the same time, the pros and cons of facebook use among the health science students has attracted the attention of educators. The data regarding its use and the effect on the life of Nepalese health science students has not yet been documented. Therefore, this study is carried out to evaluate the effect of facebook use on social interactions, behaviour, academics, and the health of students in a medical college of Nepal.

**Results:**

A cross-sectional descriptive study conducted among medical, dental, nursing and allied health science students using self-administered questionnaire. The study showed that 98.2 % of participants were facebook users. Among 452 respondents, 224 and 228 were male and female respectively, with a mean age of 20.2 ± 1.2 years. The main reason for using facebook was to remain in contact with family and friend (32 %), while its use for the academic purpose was only 5 %. However, 80.8 % of students acknowledged ease in acquiring academic materials from others, through facebook. One-fourth of the students acknowledged that they are using facebook late at night on a regular basis, while surprisingly 4.2 % of the students admitted accessing facebook during the classroom lectures. Almost two-third of the users, further admitted that facebook has had a negative impact on their studies. Burning eyes (21 %), disturbed sleep (19 %), and headache (16 %) were the most common adverse health effects reported by the facebook users. Many students (71.4 %) tried and most of them (68.7 %) succeeded, in reducing time spent on facebook, to allow for increased time devoted to their studies.

**Conclusion:**

The widespread use of facebook among the health science students, was found to have both positive and negative effects on their academics, social life, and health.

## Background

The internet has emerged as the most effective means of disseminating information. It is worth mentioning that social networking platforms such as facebook, has been in use since its beginning. Initially created for Harvard University students in 2004, as a method of socializing, this forum was opened for use by the general public in 2006. Facebook is now one of the best known social networking sites used by people of all ages and professions. It is accessible through computer, laptop, and small portable devices (tablets and cellular phones). Facebook easily allows its users to set up and maintain personal ‘profile’ pages for the purpose of connecting, interacting, and sharing personal views and content with other individuals, groups and communities online [1–3]. On average, there were 1.01 billion daily active users globally by September 2015 [4]. The use of social networking websites by those in the field of medical education has significantly increased and attracted substantial interest among educators and institutions [5]. A recent study in the United States showed that up to 96 % of medical students regularly use facebook. Facebook can be useful for students’ social life as well as their academic pursuits [6].

Facebook use by students has specific pros and cons. Particularly, the excessive use of online social media may contribute to misuse, dependence, and addictive behaviours [7]. Some studies have reviewed the impact of online social media use on mental health. These effects include changes in self-esteem [8], sleep disorders, and high percentage of depression among students [9]. However, these students were unaware of such adverse effects [10]. With the increased accessibility and availability of the internet, use of social media is on the rise in Nepal. Currently there are approximately 4.4 million facebook users [11]. To best of our knowledge, there is insufficient data regarding the use and effect of facebook on health science students in Nepal. We have hypothesized that the use of facebook may have a negative impact on the life of health science students. This study was performed to evaluate the effects of facebook on social interaction, behaviour, academics, and the health of students at Chitwan Medical College, Nepal.

## Methods

A cross-sectional study was conducted in Chitwan Medical College, Bharatpur, Nepal from September to October, 2015. The participants were medical, dental, nursing, and allied health science undergraduate students who consented to participate in the study. A self-administered questionnaire was distributed to total of 476 participants (all of the available students in basic science courses). Exclusion criteria included those who had incompletely completed the questionnaire, did not have facebook account, or had a facebook account in the past but had closed it, students complaining of illness, and anyone taking any medication. A pre-study questionnaire was given to ten students, who were not included in the study.

### Instruments

The questionnaire contained four different sections. The first section of the questionnaire included demographic characteristics such as age, gender and course of study. The second section contained six questions about the purpose and pattern of facebook use such as place, time, duration and type of device used. The third section contained three questions regarding the academic use of facebook to include its impact on study, accessibility of relevant materials, and the extent of help received in acquiring academic materials from others who are using facebook. Lastly, the fourth section contained seven questions regarding the social, psychological, and health impacts of facebook users. These included any complaint from family and friends of the students regarding excessive time spent on social media, whether or not facebook was a source of inspiration or motivation, or if the users developed any physical symptoms such as headache, eye irritation, or other symptoms following its use. The last section of the questionnaire also included a question about whether or not the student had tried to reduce the amount of time spent on facebook use and why.

### Statistical analysis

All the data was entered in Epidata 3.1, exported and analyzed through Statistical Package of Social Sciences (SPSS) (version 20). The descriptive statistical analysis of data was performed to determine the mean, standard deviation, frequency, and percentage.

## Results

Four hundred sixty-five out of 476 students returned the questionnaire (response rate—97.7 %). Five students incompletely completed the questionnaire and 8 students were not having a current facebook account. Based on our exclusion criteria, those 13 questionnaires were rejected and the remaining 452 completed questionnaires by facebook users, were subjected to analysis.

### Demographics

The number of male and female participants were 224 (49.6 %) and 228 (50.4 %) respectively. Their ages ranged from 17 to 25 years with a mean age of 20.2 ± 1.2 years. The participants from the Bachelor of Medicine and Bachelor of Surgery (MBBS) 1st year, MBBS 2nd year, Bachelor of Dental Surgery (BDS) 1st year, BDS 2nd year, Bachelor of Nursing and Allied courses (Bachelor of Pharmacy-B Pharm and Bachelor in Medical Laboratory Technology-BMLT) were 31.9, 27.9, 8.6, 8.6, 12.8 and 10.2 % respectively.

### Reasons and pattern of facebook usage

**Fig. 1:**
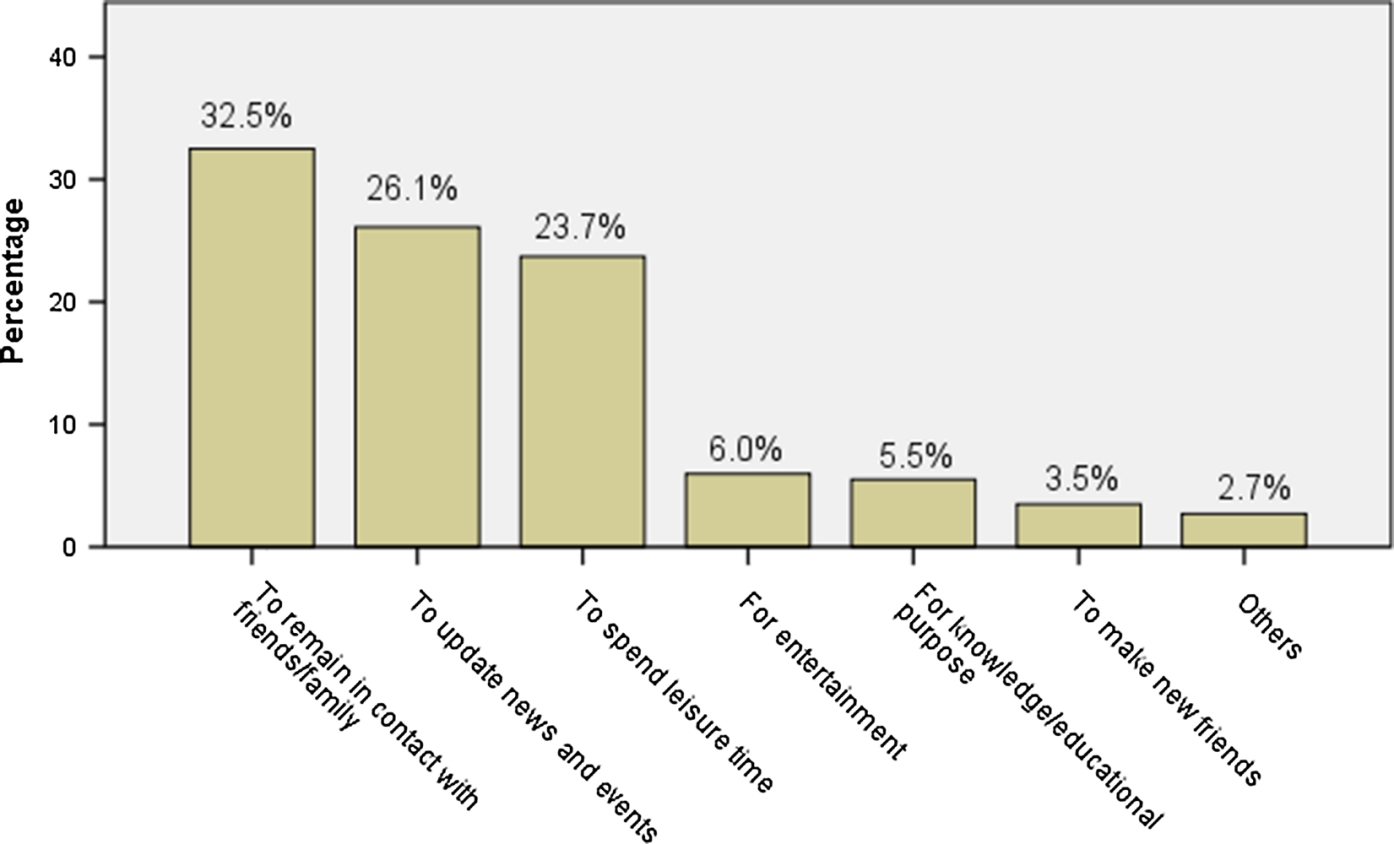
Reasons for using facebook by the participants (N = 452)

**Fig. 2:**
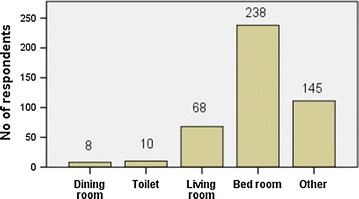
Common places in home used to surf facebook (N = 435)

**Fig. 3:**
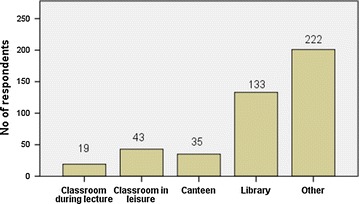
Common sites in college used to surf facebook (N = 431)

The students were found to be using facebook mainly to remain in contact with their family and friends (32 %), to get news updates (26 %) and to spend leisure time (24 %) (Fig. 1). However, only a few students (5 %) admitted to using facebook for academic purpose. Table 1 describes the pattern of facebook usage. A majority of students (84.1 %) accessed facebook on a daily basis while 44.9 % of them spent less than 1 h daily on facebook. One-fourth of the students accepted indulging in facebook until late night frequently, while 61.5 % admitted to doing so occasionally. The use of mobile devices was more common (76.5 %) among the participants than desktop/laptop for facebook use. A majority of students (54.6 %) usually accessed facebook while at their hostel. Among the majority of participants (52.7 %) the bedroom at the student’s home was the favorite place to access facebook, while 29.4 % of students used the library at college for surfing facebook. Interestingly, 4.2 % of the students admitted to accessing facebook during lecture sessions, while most of the participants (51.5 %) also accessed facebook in other locations (like playground, corridor etc.) which were not mentioned in the list of locations in the questionnaire (Figs. 2, 3).

**Table 1:** Pattern of facebook usage among participants

N = 452 (percentage)	
Do you use facebook daily?	
Yes	380 (84.1 %)
No	72 (15.9 %)
How many hours do you use facebook in a usual day? (h)	
Less than 1	203 (44.9 %)
1–2	157 (34.7 %)
2–3	53 (11.7 %)
More than 3	39 (8.6 %)
How often do you use facebook late night (after 10.00 p.m.)?	
Never	61 (13.5 %)
Sometimes	278 (61.5 %)
Most of the times	113 (25.0 %)
In a usual day, where do you use facebook mostly?	
Home	172 (38.1 %)
Hostel	247 (54.6 %)
College	22 (4.9 %)
Cafeteria	11 (2.4 %)
What do you commonly use to access facebook?	
Desktop/laptop	106 (23.5 %)
Portable devices (E.g. mobile)	346 (76.5 %)

### Academic usage of facebook and its impact on study

**Fig. 4:**
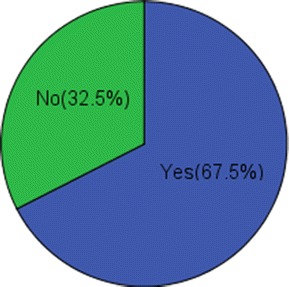
Overall negative effects of facebook on studies

A limited number of students (10.8 %) admitted to receiving relevant information for their studies directly from facebook. Conversely, 80.8 % of students acknowledged receiving academic materials from other students via facebook (Table 2). However, 67.5 % of participants noted that the facebook had a negative effect on their studies (Fig. 4).

**Table 2:** Effect of facebook use on the study

N = 452 (percentage)	
How much of the relevant information for your study is accessible through Facebook?	
Quite a lot	49 (10.8 %)
Somewhat	322 (71.2 %)
Not at all	81 (17.9 %)
To what extent do you get help in academics from others using facebook?	
Quite a lot	70 (15.5 %)
Somewhat	295 (65.3 %)
Not at all	87 (19.2 %)
Does facebook have negative effects on your study?	
Quite a lot	84 (18.6 %)
Somewhat	221 (48.9 %)
Not at all	147 (32.5 %)

### Social, psychological and health effect of facebook

**Fig. 5:**
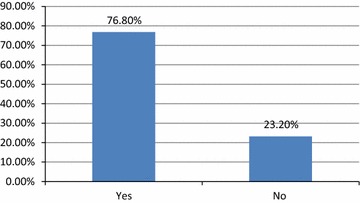
Overall negative effect of facebook on health and behaviour

A majority of the students (66.4 %) socialized more actively in real life situation than in facebook (Table 3). Some students (17.5 %) indicated that facebook was a source of inspiration and motivation for them. The study also revealed that 86.7 % of users indicated that they were annoyed when anyone disturbed them during facebook use. Among the respondents, 13.5 % reported that their friends and family frequently complained about the amount of time spent on facebook while 24.3 % of participants in the study admitted that they found it difficult to not log onto facebook during the course of an entire day. Most of the students (76.8 %) believed that facebook had negatively affected their health and behaviour (Fig. 5). The adverse health effects experienced by the participants were burning eyes (21 %), followed by disturbed sleep (19 %), headache (16 %), and others (Table 4). A majority (71.4 %) of the students admitted to making efforts to reduce their duration of facebook use and to instead use the time for the academic and creative purposes (16.2 %) (Table 3). Among these participants, 68.7 % were successful at reducing the amount of facebook use to pursue their academic goals.

**Table 3:** Effect of facebook use on personal, familial and social life

N = 452 (percentage)	
Are you more socially active on facebook than in real life?	
More active on facebook	39 (8.6 %)
More active in real	300 (66.4 %)
Equally active on both	113 (25.0 %)
Do you think facebook is a source of inspiration and motivation for you?	
Quite a lot	79 (17.5 %)
Somewhat	271 (60.0 %)
Not at all	102 (22.5 %)
Do you get irritated when anyone disturbs you while using facebook?	
Quite a lot	155 (34.3 %)
Somewhat	237 (52.4 %)
Not at all	60 (13.3 %)
How often do your friends and family complain you about the time period you spend on facebook?	
Quite a lot	61 (13.5 %)
Somewhat	214 (47.3 %)
Not at all	177 (39.2 %)
Do you feel difficult to spend your day if you can’t log into facebook for an entire day?	
Quite a lot	110 (24.3 %)
Somewhat	40 (8.8 %)
Not at all	302 (66.8 %)
Did you ever try to reduce time on facebook?	
No, I didn’t	129 (28.5 %)
Yes, I tried but couldn’t succeed	101 (22.3 %)
Yes, I tried and succeed	222 (49.1 %)
For what reason you have tried to reduce time on facebook? (N = 323)	
To use my time in academic	204 (45.1 %)
To save my time for creative work	73 (16.2 %)
To improve my relation	9 (2.0 %)
Others	37 (8.2 %)

**Table 4:** Adverse health effects on facebook users

Adverse effects	N = 452 (percentage)
Burning eyes	95 (21.0 %)
Disturbed sleep	86 (19.0 %)
Headache	73 (16.2 %)
Neck pain	33 (7.3 %)
Back pain	22 (4.9 %)
Reduced appetite	4 (0.9 %)
Others	34 (7.5 %)
None	105 (23.2 %)

## Discussion

This study demonstrated the widespread use of facebook among 98.2 % of the health science students in a medical college of Nepal. This was found to be similar in comparison, to studies conducted in different countries [12–14] on the same subject. In addition to ease of connecting with friends or family, facebook’s news updates in a variety of different categories (sports, politics, education, health etc.) encourages its users to spend even more of their leisure time on the site. The increasing availability of Wi-Fi (wireless fidelity), portable devices, and the popularity of facebook itself, might have also contributed to facebook use among this population. According to Hew KF [2], facebook has very little educational value and the participants mainly used facebook to keep in contact with the known individuals which were found to be concurring with this study. Moreover, this study also demonstrated that only 5 % of participants used facebook for academic purpose, while other studies conducted by Raacke et al. [15] and Gray et al. [13] reported academic use to be higher (10.9 and 25.5 % respectively). This difference could be due to academic culture and varied institutional policies on the use of internet in these institutions. While one-fourth of users in this study were found to be accessing facebook during leisure time, when accessed regularly, there is potential risk for developing addictive behaviours, through the development of poor self-discipline and task avoidance [16].

A majority of the students accessed facebook on daily basis, in higher numbers than similar past studies [13, 17]. Most of the students spent time on facebook on average, less than 1–2 h daily, which was consistent with the findings reported by others [18, 19]. A majority of participants in this study admitted that they were on facebook until late at night (61.5 %). A similar finding was obtained by Farooqi et al. among the students of Dow University in Pakistan [20]. Young KS found that the university students’ sleep patterns were disrupted due to facebook use late at night, leading to fatigue and impaired academic performance [21]. This study also supported the findings of previous studies [6, 17] that the majority of students accessed facebook using mobile devices. This particular method of accessing facebook could be due to increased availability of internet access and newer versions of operating systems on mobile devices that support facebook use.

Similar to the previous studies [2, 6, 22], a small number of students reported that relevant academic information was accessible through the facebook. The majority of the participants felt that they could acquire academic assistance from other facebook users to some degree, which was higher than a previous study conducted by Gafni and Deri [6]. Facebook allows for rapid, easy access, and immediate interaction among students and their teachers. This use of facebook can facilitate rapid consultation and dissemination of lecture notes, prior exam information, and other information, much more quickly than using traditional methods of learning. As a result, facebook has been found to be effective in obtaining information quickly from others, saving time, and money [6]. However, Hew [2] reviewed that only a minimal number of facebook users actually asked for assistance from facebook friends, while most of the students preferred discussing their personal life over their studies. Therefore, spending time on facebook may significantly compromise a student’s academic success. In this study, 67.5 % of the users admitted that facebook negatively affected their studies, which was higher than the finding of a previous study by Farooqi et al. [20].

The data from previous studies clearly indicated that the excessive use of social media, compromises actual live social interaction and academic accomplishments. It may also be associated with relationship problems, personal loneliness, and depression [23, 24]. We found that very small population (8.6 %) spent more time on facebook than live interactions with others, unlike the result obtained by the previous study [20]. However, 17.4 % of participants indicated that facebook was a source of inspiration and motivation for them. This is consistent with the findings of Farooqi et al. who observed that many students admitted that excessive facebook use had ruined their social life, causing them to spend less time with their loved ones [20]. Moreover, the researchers at the University of Southern California reported that an increasing number of people, admitted to spending less time with their family members as result of excessive use of the internet, including social networking sites [25].

Young people have an increased tendency to develop additive behaviour with facebook use. However, they are usually unaware of this additive behaviour [20]. In this study, one-fourth of the users found it difficult to spend an entire day without accessing facebook. Half of them complained of feeling irritated when they were disturbed while using facebook, which was consistent with the findings of previous studies [17, 20]. More than half of the participants received complaints from their family or friends about the amount of time they spent on facebook. As these are some of the key components in determining the facebook addiction according to ‘Bergen Facebook Addiction Scale (BFAS)’ [26], this supports the theory that these participants possibly have some degree of facebook addiction. Sharifah et al. identified the negative behavioural consequences of social networking as: hyperactivity, attention deficit, depression, and multi-tasking mania [27]. Thus, proper education regarding the use of social media is needed.

As with any other technology facebook is not unique for eliciting both positive and negative effects on its users. Previous studies have shown adverse effects similar to computer use, such as: headache, backache, weight change, and eye problems [17, 28]. Of those reported by the users in this study, burning eyes, disturbed sleep, and headache were the most common reported adverse health effects. On the other hand, 23.2 % of participants denied experiencing any adverse effects. Sierra et al. stated that both quantity and quality of sleep might strongly influence mood [29] and subjective well-being, which in turn could impact the academic performance [21, 30] of the students who use facebook late at night and do not allow for sufficient sleep. Al-Dubai et al. had clearly indicated a significant association between facebook use, its adverse health effects, and unhealthy behavior. They have recommended that higher educational institutions should create awareness and safer practices for their students. Additionally, they have advocated regular health screening of students to avoid the possible health consequences due to facebook use [31].

Although a majority of students (71.5 %) claimed that they tried to reduce their time spent on facebook, one-fourth of them were not successful indicating additive behavior, consistent with the result found by Farooqi et al. [20]. It is worth mentioning that the students were willing to invest more of their time on their studies rather than on facebook. Further studies in a large population may reveal the factors associated with positive and negative impacts of facebook use among the health science students.

Since information technology is rapidly evolving, popular social networks like facebook, could be used by the educational institutions for academic purposes, such as uploading data and sharing educational materials. It is likely that more students will use social sites like facebook in future, as more attractive features are offered to their users. This will undoubtedly affect the students’ learning process and have an impact on their health and personal life. Therefore, new approaches and tools need to be developed for evaluating the facebook use and its consequences among students.

## Conclusions

Excessive use of facebook, a popular social network site, has important positive and negative effects on the academics, social interactions, and health of the health science students in this study. With limited academic benefits, excessive use of facebook may actually increase the risk of facebook additive behavior, resulting in less time spent on academics. It is important to involve the parents of students, educational institutions, and the facebook authority, to collaborate on how to encourage students to limit social media usage and bring awareness to the consequences of excessive use, especially among the student population.

### Limitations of the study

Since this is a cross-sectional study conducted in a single medical school in Nepal, this study might not be representative of all health science students. We believe that similar studies are important in exploring and understanding the practices of facebook and other social media use along with its consequences. The results of this study revealed that the health problems were prevalent in facebook users. However, the specific cause and effects could be better established through further studies. Future research could be designed to explore the effects of excessive use of different social media among students in different academic programs.

## Abbreviations

SPSS: statistical package for the social sciences
MBBS: Bachelor of Medicine and Bachelor of Surgery
BDS: Bachelor of Dental Surgery
B Pharm: Bachelor of Pharmacy
BMLT: Bachelor in Medical Laboratory Technology
